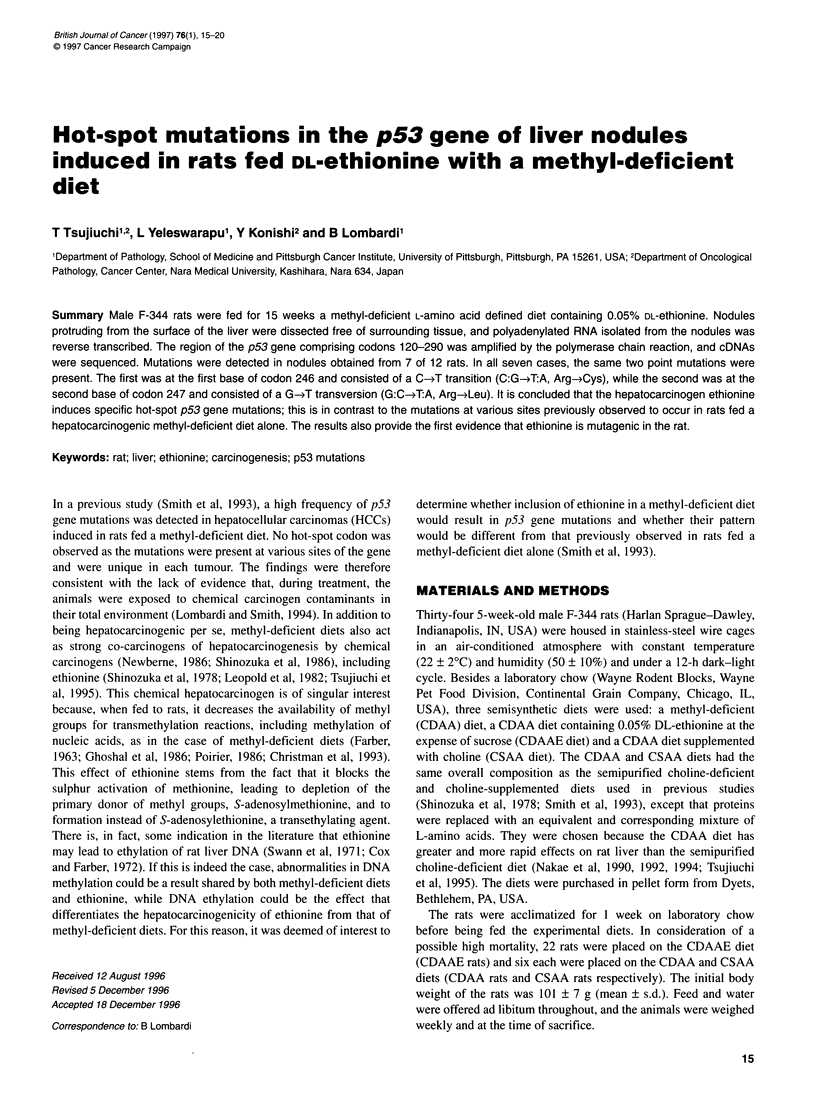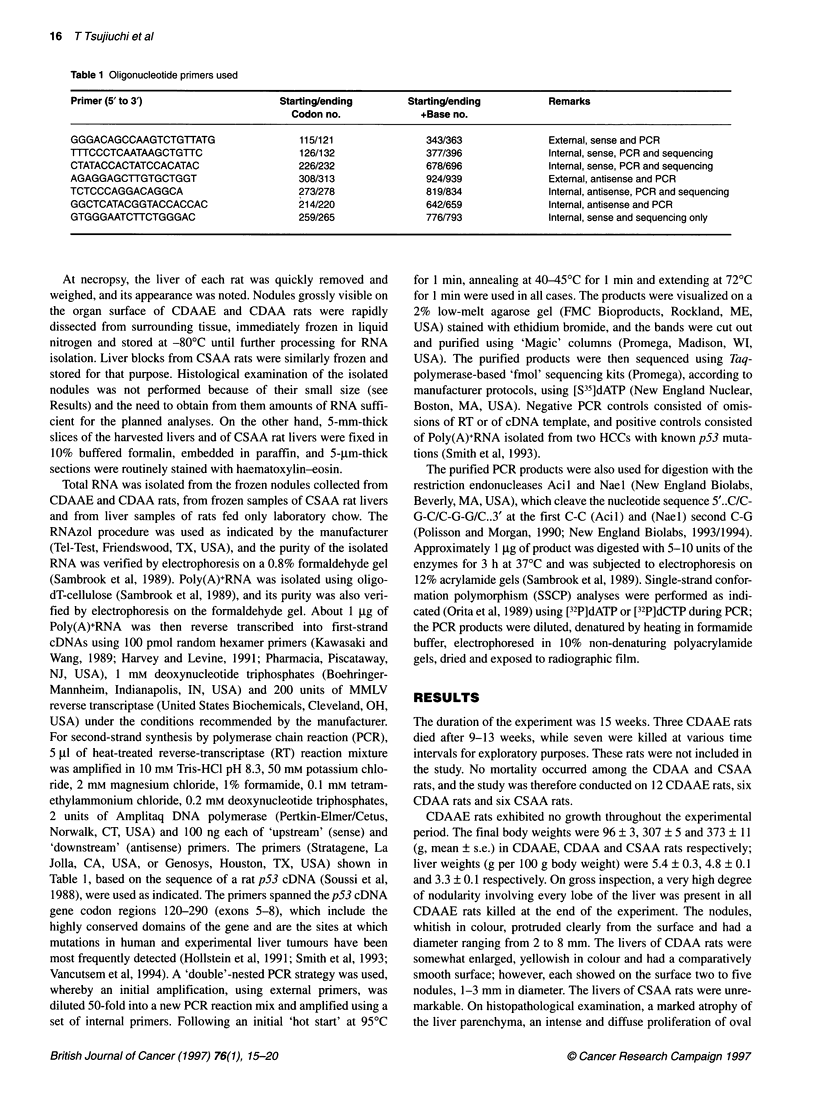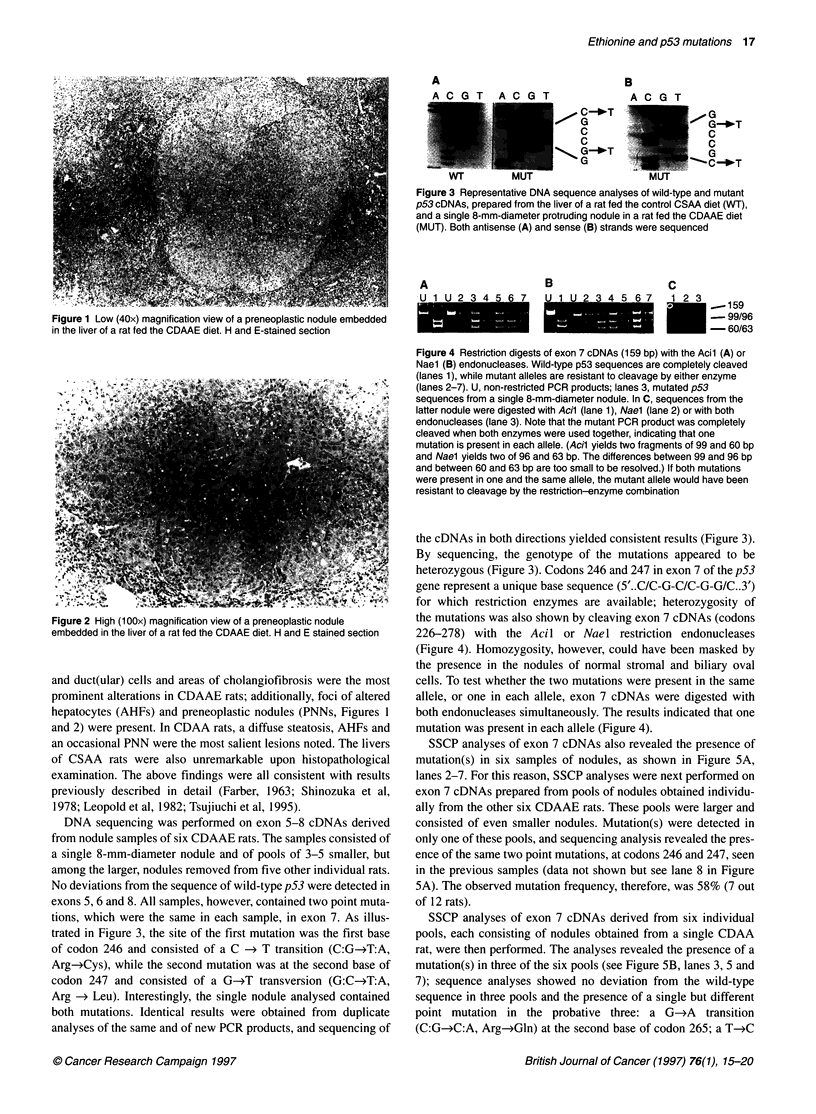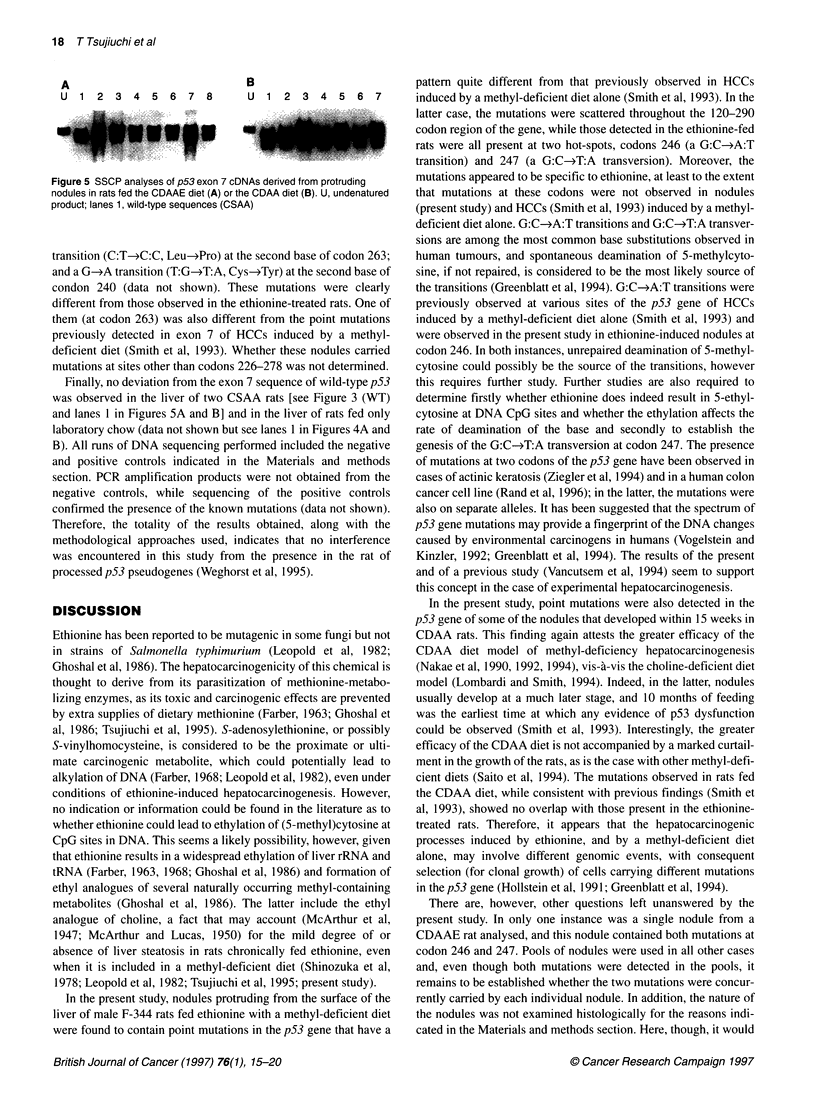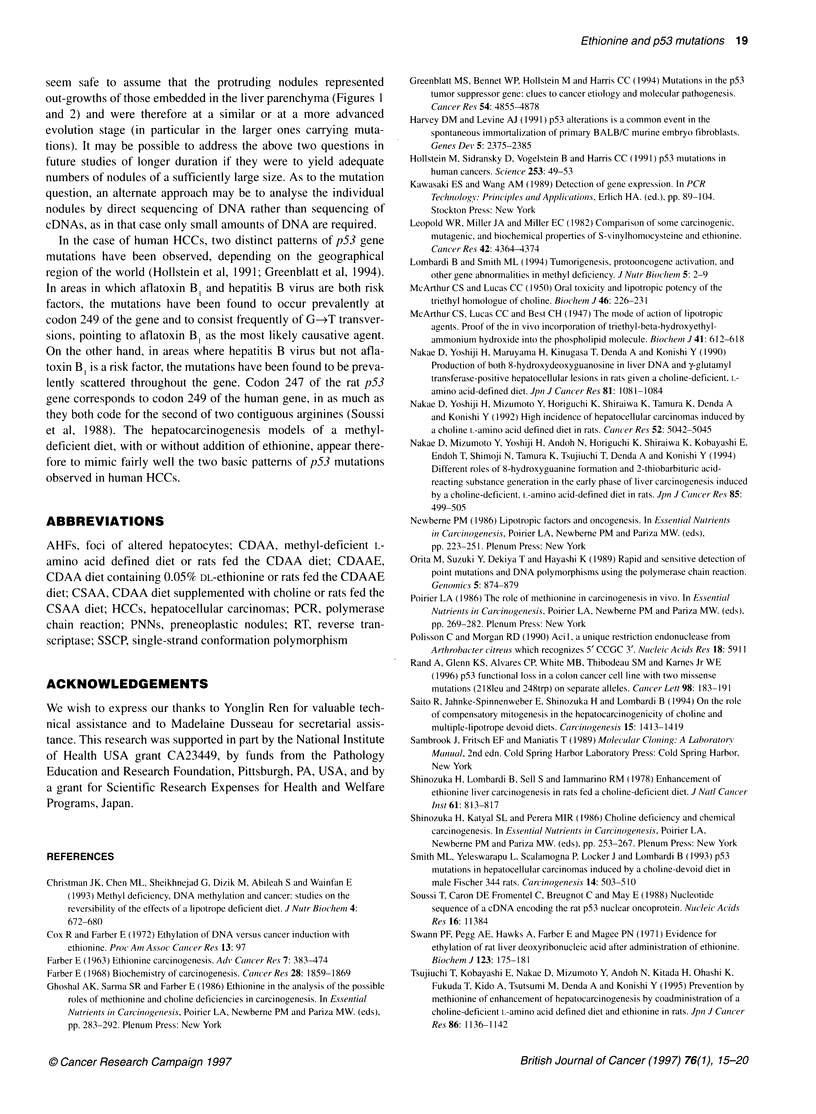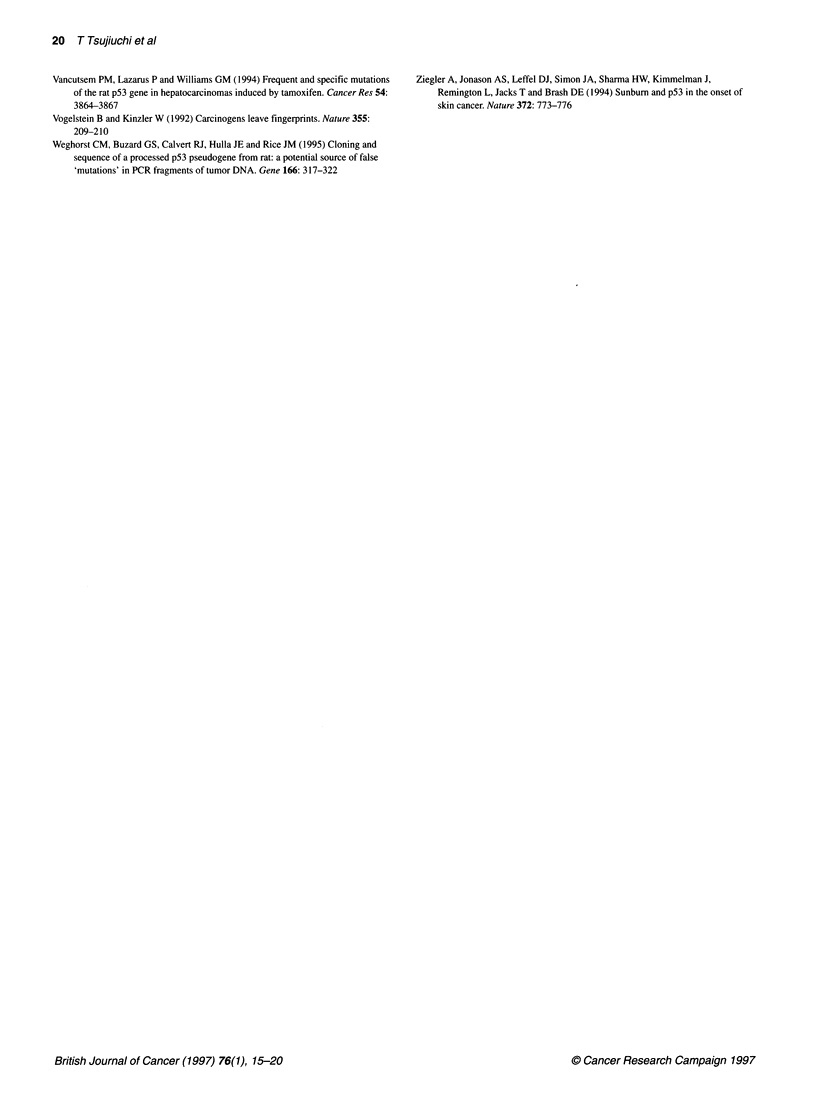# Hot-spot mutations in the p53 gene of liver nodules induced in rats fed DL-ethionine with a methyl-deficient diet.

**DOI:** 10.1038/bjc.1997.329

**Published:** 1997

**Authors:** T. Tsujiuchi, L. Yeleswarapu, Y. Konishi, B. Lombardi

**Affiliations:** Department of Pathology, School of Medicine and Pittsburgh Cancer Institute, University of Pittsburgh, PA 15261, USA.

## Abstract

**Images:**


British Journal of Cancer (1997) 76(1), 15–20
© 1997 Cancer Research Campaign

### Hot-spot mutations in the *p53* gene of liver nodules induced in rats fed DL-ethionine with a methyl-deficient diet

**T Tsujiuchi[1,2], L Yeleswarapu[1], Y Konishi[2] and B Lombardi[1]**

[1]Department of Pathology, School of Medicine and Pittsburgh Cancer Institute, University of Pittsburgh, Pittsburgh, PA 15261, USA; [2]Department of Oncological Pathology, Cancer Center, Nara Medical University, Kashihara, Nara 634, Japan

**Summary** Male F-344 rats were fed for 15 weeks a methyl-deficient L-amino acid defined diet containing 0.05% DL-ethionine. Nodules protruding from the surface of the liver were dissected free of surrounding tissue, and polyadenylated RNA isolated from the nodules was reverse transcribed. The region of the *p53* gene comprising codons 120–290 was amplified by the polymerase chain reaction, and cDNAs were sequenced. Mutations were detected in nodules obtained from 7 of 12 rats. In all seven cases, the same two point mutations were present. The first was at the first base of codon 246 and consisted of a C→T transition (C:G→T:A, Arg→Cys), while the second was at the second base of codon 247 and consisted of a G→T transversion (G:C→T:A, Arg→Leu). It is concluded that the hepatocarcinogen ethionine induces specific hot-spot *p53* gene mutations; this is in contrast to the mutations at various sites previously observed to occur in rats fed a hepatocarcinogenic methyl-deficient diet alone. The results also provide the first evidence that ethionine is mutagenic in the rat.

**Keywords:** rat; liver; ethionine; carcinogenesis; p53 mutations

In a previous study (Smith et al, 1993), a high frequency of *p53* gene mutations was detected in hepatocellular carcinomas (HCCs) induced in rats fed a methyl-deficient diet. No hot-spot codon was observed as the mutations were present at various sites of the gene and were unique in each tumour. The findings were therefore consistent with the lack of evidence that, during treatment, the animals were exposed to chemical carcinogen contaminants in their total environment (Lombardi and Smith, 1994). In addition to being hepatocarcinogenic per se, methyl-deficient diets also act as strong co-carcinogens of hepatocarcinogenesis by chemical carcinogens (Newberne, 1986; Shinozuka et al, 1986), including ethionine (Shinozuka et al, 1978; Leopold et al, 1982; Tsujiuchi et al, 1995). This chemical hepatocarcinogen is of singular interest because, when fed to rats, it decreases the availability of methyl groups for transmethylation reactions, including methylation of nucleic acids, as in the case of methyl-deficient diets (Farber, 1963; Ghoshal et al, 1986; Poirier, 1986; Christman et al, 1993). This effect of ethionine stems from the fact that it blocks the sulphur activation of methionine, leading to depletion of the primary donor of methyl groups, *S*-adenosylmethionine, and to formation instead of *S*-adenosylethionine, a transethylating agent. There is, in fact, some indication in the literature that ethionine may lead to ethylation of rat liver DNA (Swann et al, 1971; Cox and Farber, 1972). If this is indeed the case, abnormalities in DNA methylation could be a result shared by both methyl-deficient diets and ethionine, while DNA ethylation could be the effect that differentiates the hepatocarcinogenicity of ethionine from that of methyl-deficient diets. For this reason, it was deemed of interest to determine whether inclusion of ethionine in a methyl-deficient diet would result in *p53* gene mutations and whether their pattern would be different from that previously observed in rats fed a methyl-deficient diet alone (Smith et al, 1993).

*Received 12 August 1996*
*Revised 5 December 1996*
*Accepted 18 December 1996*

*Correspondence to:* B Lombardi

### MATERIALS AND METHODS

Thirty-four 5-week-old male F-344 rats (Harlan Sprague–Dawley, Indianapolis, IN, USA) were housed in stainless-steel wire cages in an air-conditioned atmosphere with constant temperature (22 ± 2°C) and humidity (50 ± 10%) and under a 12-h dark–light cycle. Besides a laboratory chow (Wayne Rodent Blocks, Wayne Pet Food Division, Continental Grain Company, Chicago, IL, USA), three semisynthetic diets were used: a methyl-deficient (CDAA) diet, a CDAA diet containing 0.05% DL-ethionine at the expense of sucrose (CDAAE diet) and a CDAA diet supplemented with choline (CSAA diet). The CDAA and CSAA diets had the same overall composition as the semipurified choline-deficient and choline-supplemented diets used in previous studies (Shinozuka et al, 1978; Smith et al, 1993), except that proteins were replaced with an equivalent and corresponding mixture of L-amino acids. They were chosen because the CDAA diet has greater and more rapid effects on rat liver than the semipurified choline-deficient diet (Nakae et al, 1990, 1992, 1994; Tsujiuchi et al, 1995). The diets were purchased in pellet form from Dyets, Bethlehem, PA, USA.

The rats were acclimatized for 1 week on laboratory chow before being fed the experimental diets. In consideration of a possible high mortality, 22 rats were placed on the CDAAE diet (CDAAE rats) and six each were placed on the CDAA and CSAA diets (CDAA rats and CSAA rats respectively). The initial body weight of the rats was 101 ± 7 g (mean ± s.d.). Feed and water were offered ad libitum throughout, and the animals were weighed weekly and at the time of sacrifice.

**Table 1** Oligonucleotide primers used

| Primer (5′ to 3′) | Starting/ending Codon no. | Starting/ending +Base no. | Remarks |
|---|---|---|---|
| GGGACAGCCAAGTCTGTTATG | 115/121 | 343/363 | External, sense and PCR |
| TTTCCCTCAATAAGCTGTTC | 126/132 | 377/396 | Internal, sense, PCR and sequencing |
| CTATACCACTATCCACATAC | 226/232 | 678/696 | Internal, sense, PCR and sequencing |
| AGAGGAGCTTGTGCTGGT | 308/313 | 924/939 | External, antisense and PCR |
| TCTCCCAGGACAGGCA | 273/278 | 819/834 | Internal, antisense, PCR and sequencing |
| GGCTCATACGGTACCACCAC | 214/220 | 642/659 | Internal, antisense and PCR |
| GTGGGAATCTTCTGGGAC | 259/265 | 776/793 | Internal, sense and sequencing only |

At necropsy, the liver of each rat was quickly removed and weighed, and its appearance was noted. Nodules grossly visible on the organ surface of CDAAE and CDAA rats were rapidly dissected from surrounding tissue, immediately frozen in liquid nitrogen and stored at –80°C until further processing for RNA isolation. Liver blocks from CSAA rats were similarly frozen and stored for that purpose. Histological examination of the isolated nodules was not performed because of their small size (see Results) and the need to obtain from them amounts of RNA sufficient for the planned analyses. On the other hand, 5-mm-thick slices of the harvested livers and of CSAA rat livers were fixed in 10% buffered formalin, embedded in paraffin, and 5-μm-thick sections were routinely stained with haematoxylin–eosin.

Total RNA was isolated from the frozen nodules collected from CDAAE and CDAA rats, from frozen samples of CSAA rat livers and from liver samples of rats fed only laboratory chow. The RNAzol procedure was used as indicated by the manufacturer (Tel-Test, Friendswood, TX, USA), and the purity of the isolated RNA was verified by electrophoresis on a 0.8% formaldehyde gel (Sambrook et al, 1989). Poly(A)+RNA was isolated using oligo-dT-cellulose (Sambrook et al, 1989), and its purity was also verified by electrophoresis on the formaldehyde gel. About 1 μg of Poly(A)+RNA was then reverse transcribed into first-strand cDNAs using 100 pmol random hexamer primers (Kawasaki and Wang, 1989; Harvey and Levine, 1991; Pharmacia, Piscataway, NJ, USA), 1 mM deoxynucleotide triphosphates (Boehringer-Mannheim, Indianapolis, IN, USA) and 200 units of MMLV reverse transcriptase (United States Biochemicals, Cleveland, OH, USA) under the conditions recommended by the manufacturer. For second-strand synthesis by polymerase chain reaction (PCR), 5 μl of heat-treated reverse-transcriptase (RT) reaction mixture was amplified in 10 mM Tris-HCl pH 8.3, 50 mM potassium chloride, 2 mM magnesium chloride, 1% formamide, 0.1 mM tetramethylammonium chloride, 0.2 mM deoxynucleotide triphosphates, 2 units of Amplitaq DNA polymerase (Pertkin-Elmer/Cetus, Norwalk, CT, USA) and 100 ng each of 'upstream' (sense) and 'downstream' (antisense) primers. The primers (Stratagene, La Jolla, CA, USA, or Genosys, Houston, TX, USA) shown in Table 1, based on the sequence of a rat *p53* cDNA (Soussi et al, 1988), were used as indicated. The primers spanned the *p53* cDNA gene codon regions 120–290 (exons 5–8), which include the highly conserved domains of the gene and are the sites at which mutations in human and experimental liver tumours have been most frequently detected (Hollstein et al, 1991; Smith et al, 1993; Vancutsem et al, 1994). A 'double'-nested PCR strategy was used, whereby an initial amplification, using external primers, was diluted 50-fold into a new PCR reaction mix and amplified using a set of internal primers. Following an initial 'hot start' at 95°C for 1 min, annealing at 40–45°C for 1 min and extending at 72°C for 1 min were used in all cases. The products were visualized on a 2% low-melt agarose gel (FMC Bioproducts, Rockland, ME, USA) stained with ethidium bromide, and the bands were cut out and purified using 'Magic' columns (Promega, Madison, WI, USA). The purified products were then sequenced using *Taq*-polymerase-based 'fmol' sequencing kits (Promega), according to manufacturer protocols, using [$S^{35}$]dATP (New England Nuclear, Boston, MA, USA). Negative PCR controls consisted of omissions of RT or of cDNA template, and positive controls consisted of Poly(A)+RNA isolated from two HCCs with known *p53* mutations (Smith et al, 1993).

The purified PCR products were also used for digestion with the restriction endonucleases Aci1 and Nae1 (New England Biolabs, Beverly, MA, USA), which cleave the nucleotide sequence 5′..C/C-G-C/C-G-G/C..3′ at the first C-C (Aci1) and (Nae1) second C-G (Polisson and Morgan, 1990; New England Biolabs, 1993/1994). Approximately 1 μg of product was digested with 5–10 units of the enzymes for 3 h at 37°C and was subjected to electrophoresis on 12% acrylamide gels (Sambrook et al, 1989). Single-strand conformation polymorphism (SSCP) analyses were performed as indicated (Orita et al, 1989) using [$^{32}$P]dATP or [$^{32}$P]dCTP during PCR; the PCR products were diluted, denatured by heating in formamide buffer, electrophoresed in 10% non-denaturing polyacrylamide gels, dried and exposed to radiographic film.

### RESULTS

The duration of the experiment was 15 weeks. Three CDAAE rats died after 9–13 weeks, while seven were killed at various time intervals for exploratory purposes. These rats were not included in the study. No mortality occurred among the CDAA and CSAA rats, and the study was therefore conducted on 12 CDAAE rats, six CDAA rats and six CSAA rats.

CDAAE rats exhibited no growth throughout the experimental period. The final body weights were 96 ± 3, 307 ± 5 and 373 ± 11 (g, mean ± s.e.) in CDAAE, CDAA and CSAA rats respectively; liver weights (g per 100 g body weight) were 5.4 ± 0.3, 4.8 ± 0.1 and 3.3 ± 0.1 respectively. On gross inspection, a very high degree of nodularity involving every lobe of the liver was present in all CDAAE rats killed at the end of the experiment. The nodules, whitish in colour, protruded clearly from the surface and had a diameter ranging from 2 to 8 mm. The livers of CDAA rats were somewhat enlarged, yellowish in colour and had a comparatively smooth surface; however, each showed on the surface two to five nodules, 1–3 mm in diameter. The livers of CSAA rats were unremarkable. On histopathological examination, a marked atrophy of the liver parenchyma, an intense and diffuse proliferation of oval

 © Cancer Research Campaign 1997

**Figure 1** Low (40×) magnification view of a preneoplastic nodule embedded in the liver of a rat fed the CDAAE diet. H and E-stained section

**Figure 2** High (100×) magnification view of a preneoplastic nodule embedded in the liver of a rat fed the CDAAE diet. H and E stained section

**Figure 3** Representative DNA sequence analyses of wild-type and mutant *p53* cDNAs, prepared from the liver of a rat fed the control CSAA diet (WT), and a single 8-mm-diameter protruding nodule in a rat fed the CDAAE diet (MUT). Both antisense (**A**) and sense (**B**) strands were sequenced

**Figure 4** Restriction digests of exon 7 cDNAs (159 bp) with the Aci1 (**A**) or Nae1 (**B**) endonucleases. Wild-type p53 sequences are completely cleaved (lanes 1), while mutant alleles are resistant to cleavage by either enzyme (lanes 2–7). U, non-restricted PCR products; lanes 3, mutated *p53* sequences from a single 8-mm-diameter nodule. In **C**, sequences from the latter nodule were digested with *Aci*1 (lane 1), *Nae*1 (lane 2) or with both endonucleases (lane 3). Note that the mutant PCR product was completely cleaved when both enzymes were used together, indicating that one mutation is present in each allele. (*Aci*1 yields two fragments of 99 and 60 bp and *Nae*1 yields two of 96 and 63 bp. The differences between 99 and 96 bp and between 60 and 63 bp are too small to be resolved.) If both mutations were present in one and the same allele, the mutant allele would have been resistant to cleavage by the restriction–enzyme combination and duct(ular) cells and areas of cholangiofibrosis were the most prominent alterations in CDAAE rats; additionally, foci of altered hepatocytes (AHFs) and preneoplastic nodules (PNNs, Figures 1 and 2) were present. In CDAA rats, a diffuse steatosis, AHFs and an occasional PNN were the most salient lesions noted. The livers of CSAA rats were also unremarkable upon histopathological examination. The above findings were all consistent with results previously described in detail (Farber, 1963; Shinozuka et al, 1978; Leopold et al, 1982; Tsujiuchi et al, 1995).

DNA sequencing was performed on exon 5–8 cDNAs derived from nodule samples of six CDAAE rats. The samples consisted of a single 8-mm-diameter nodule and of pools of 3–5 smaller, but among the larger, nodules removed from five other individual rats. No deviations from the sequence of wild-type *p53* were detected in exons 5, 6 and 8. All samples, however, contained two point mutations, which were the same in each sample, in exon 7. As illustrated in Figure 3, the site of the first mutation was the first base of codon 246 and consisted of a C → T transition (C:G→T:A, Arg→Cys), while the second mutation was at the second base of codon 247 and consisted of a G→T transversion (G:C→T:A, Arg → Leu). Interestingly, the single nodule analysed contained both mutations. Identical results were obtained from duplicate analyses of the same and of new PCR products, and sequencing of the cDNAs in both directions yielded consistent results (Figure 3). By sequencing, the genotype of the mutations appeared to be heterozygous (Figure 3). Codons 246 and 247 in exon 7 of the *p53* gene represent a unique base sequence (5′..C/C-G-C/C-G-G/C..3′) for which restriction enzymes are available; heterozygosity of the mutations was also shown by cleaving exon 7 cDNAs (codons 226–278) with the *Aci*l or *Nae*l restriction endonucleases (Figure 4). Homozygosity, however, could have been masked by the presence in the nodules of normal stromal and biliary oval cells. To test whether the two mutations were present in the same allele, or one in each allele, exon 7 cDNAs were digested with both endonucleases simultaneously. The results indicated that one mutation was present in each allele (Figure 4).

SSCP analyses of exon 7 cDNAs also revealed the presence of mutation(s) in six samples of nodules, as shown in Figure 5A, lanes 2–7. For this reason, SSCP analyses were next performed on exon 7 cDNAs prepared from pools of nodules obtained individually from the other six CDAAE rats. These pools were larger and consisted of even smaller nodules. Mutation(s) were detected in only one of these pools, and sequencing analysis revealed the presence of the same two point mutations, at codons 246 and 247, seen in the previous samples (data not shown but see lane 8 in Figure 5A). The observed mutation frequency, therefore, was 58% (7 out of 12 rats).

SSCP analyses of exon 7 cDNAs derived from six individual pools, each consisting of nodules obtained from a single CDAA rat, were then performed. The analyses revealed the presence of a mutation(s) in three of the six pools (see Figure 5B, lanes 3, 5 and 7); sequence analyses showed no deviation from the wild-type sequence in three pools and the presence of a single but different point mutation in the probative three: a G→A transition (C:G→C:A, Arg→Gln) at the second base of codon 265; a T→C

© Cancer Research Campaign 1997

**Figure 5** SSCP analyses of *p53* exon 7 cDNAs derived from protruding nodules in rats fed the CDAAE diet (**A**) or the CDAA diet (**B**). U, undenatured product; lanes 1, wild-type sequences (CSAA)

transition (C:T→C:C, Leu→Pro) at the second base of codon 263; and a G→A transition (T:G→T:A, Cys→Tyr) at the second base of condon 240 (data not shown). These mutations were clearly different from those observed in the ethionine-treated rats. One of them (at codon 263) was also different from the point mutations previously detected in exon 7 of HCCs induced by a methyl-deficient diet (Smith et al, 1993). Whether these nodules carried mutations at sites other than codons 226–278 was not determined.

Finally, no deviation from the exon 7 sequence of wild-type *p53* was observed in the liver of two CSAA rats [see Figure 3 (WT) and lanes 1 in Figures 5A and B] and in the liver of rats fed only laboratory chow (data not shown but see lanes 1 in Figures 4A and B). All runs of DNA sequencing performed included the negative and positive controls indicated in the Materials and methods section. PCR amplification products were not obtained from the negative controls, while sequencing of the positive controls confirmed the presence of the known mutations (data not shown). Therefore, the totality of the results obtained, along with the methodological approaches used, indicates that no interference was encountered in this study from the presence in the rat of processed *p53* pseudogenes (Weghorst et al, 1995).

### DISCUSSION

Ethionine has been reported to be mutagenic in some fungi but not in strains of *Salmonella typhimurium* (Leopold et al, 1982; Ghoshal et al, 1986). The hepatocarcinogenicity of this chemical is thought to derive from its parasitization of methionine-metabolizing enzymes, as its toxic and carcinogenic effects are prevented by extra supplies of dietary methionine (Farber, 1963; Ghoshal et al, 1986; Tsujiuchi et al, 1995). *S*-adenosylethionine, or possibly *S*-vinylhomocysteine, is considered to be the proximate or ultimate carcinogenic metabolite, which could potentially lead to alkylation of DNA (Farber, 1968; Leopold et al, 1982), even under conditions of ethionine-induced hepatocarcinogenesis. However, no indication or information could be found in the literature as to whether ethionine could lead to ethylation of (5-methyl)cytosine at CpG sites in DNA. This seems a likely possibility, however, given that ethionine results in a widespread ethylation of liver rRNA and tRNA (Farber, 1963, 1968; Ghoshal et al, 1986) and formation of ethyl analogues of several naturally occurring methyl-containing metabolites (Ghoshal et al, 1986). The latter include the ethyl analogue of choline, a fact that may account (McArthur et al, 1947; McArthur and Lucas, 1950) for the mild degree of or absence of liver steatosis in rats chronically fed ethionine, even when it is included in a methyl-deficient diet (Shinozuka et al, 1978; Leopold et al, 1982; Tsujiuchi et al, 1995; present study).

In the present study, nodules protruding from the surface of the liver of male F-344 rats fed ethionine with a methyl-deficient diet were found to contain point mutations in the *p53* gene that have a pattern quite different from that previously observed in HCCs induced by a methyl-deficient diet alone (Smith et al, 1993). In the latter case, the mutations were scattered throughout the 120–290 codon region of the gene, while those detected in the ethionine-fed rats were all present at two hot-spots, codons 246 (a G:C→A:T transition) and 247 (a G:C→T:A transversion). Moreover, the mutations appeared to be specific to ethionine, at least to the extent that mutations at these codons were not observed in nodules (present study) and HCCs (Smith et al, 1993) induced by a methyl-deficient diet alone. G:C→A:T transitions and G:C→T:A transversions are among the most common base substitutions observed in human tumours, and spontaneous deamination of 5-methylcytosine, if not repaired, is considered to be the most likely source of the transitions (Greenblatt et al, 1994). G:C→A:T transitions were previously observed at various sites of the *p53* gene of HCCs induced by a methyl-deficient diet alone (Smith et al, 1993) and were observed in the present study in ethionine-induced nodules at codon 246. In both instances, unrepaired deamination of 5-methylcytosine could possibly be the source of the transitions, however this requires further study. Further studies are also required to determine firstly whether ethionine does indeed result in 5-ethylcytosine at DNA CpG sites and whether the ethylation affects the rate of deamination of the base and secondly to establish the genesis of the G:C→T:A transversion at codon 247. The presence of mutations at two codons of the *p53* gene have been observed in cases of actinic keratosis (Ziegler et al, 1994) and in a human colon cancer cell line (Rand et al, 1996); in the latter, the mutations were also on separate alleles. It has been suggested that the spectrum of *p53* gene mutations may provide a fingerprint of the DNA changes caused by environmental carcinogens in humans (Vogelstein and Kinzler, 1992; Greenblatt et al, 1994). The results of the present and of a previous study (Vancutsem et al, 1994) seem to support this concept in the case of experimental hepatocarcinogenesis.

In the present study, point mutations were also detected in the *p53* gene of some of the nodules that developed within 15 weeks in CDAA rats. This finding again attests the greater efficacy of the CDAA diet model of methyl-deficiency hepatocarcinogenesis (Nakae et al, 1990, 1992, 1994), vis-à-vis the choline-deficient diet model (Lombardi and Smith, 1994). Indeed, in the latter, nodules usually develop at a much later stage, and 10 months of feeding was the earliest time at which any evidence of p53 dysfunction could be observed (Smith et al, 1993). Interestingly, the greater efficacy of the CDAA diet is not accompanied by a marked curtailment in the growth of the rats, as is the case with other methyl-deficient diets (Saito et al, 1994). The mutations observed in rats fed the CDAA diet, while consistent with previous findings (Smith et al, 1993), showed no overlap with those present in the ethionine-treated rats. Therefore, it appears that the hepatocarcinogenic processes induced by ethionine, and by a methyl-deficient diet alone, may involve different genomic events, with consequent selection (for clonal growth) of cells carrying different mutations in the *p53* gene (Hollstein et al, 1991; Greenblatt et al, 1994).

There are, however, other questions left unanswered by the present study. In only one instance was a single nodule from a CDAAE rat analysed, and this nodule contained both mutations at codon 246 and 247. Pools of nodules were used in all other cases and, even though both mutations were detected in the pools, it remains to be established whether the two mutations were concurrently carried by each individual nodule. In addition, the nature of the nodules was not examined histologically for the reasons indicated in the Materials and methods section. Here, though, it would

© Cancer Research Campaign 1997

seem safe to assume that the protruding nodules represented out-growths of those embedded in the liver parenchyma (Figures 1 and 2) and were therefore at a similar or at a more advanced evolution stage (in particular in the larger ones carrying mutations). It may be possible to address the above two questions in future studies of longer duration if they were to yield adequate numbers of nodules of a sufficiently large size. As to the mutation question, an alternate approach may be to analyse the individual nodules by direct sequencing of DNA rather than sequencing of cDNAs, as in that case only small amounts of DNA are required.

In the case of human HCCs, two distinct patterns of *p53* gene mutations have been observed, depending on the geographical region of the world (Hollstein et al, 1991; Greenblatt et al, 1994). In areas in which aflatoxin $B_1$ and hepatitis B virus are both risk factors, the mutations have been found to occur prevalently at codon 249 of the gene and to consist frequently of G→T transversions, pointing to aflatoxin $B_1$ as the most likely causative agent. On the other hand, in areas where hepatitis B virus but not aflatoxin $B_1$ is a risk factor, the mutations have been found to be prevalently scattered throughout the gene. Codon 247 of the rat *p53* gene corresponds to codon 249 of the human gene, in as much as they both code for the second of two contiguous arginines (Soussi et al, 1988). The hepatocarcinogenesis models of a methyl-deficient diet, with or without addition of ethionine, appear therefore to mimic fairly well the two basic patterns of *p53* mutations observed in human HCCs.

### ABBREVIATIONS

AHFs, foci of altered hepatocytes; CDAA, methyl-deficient L-amino acid defined diet or rats fed the CDAA diet; CDAAE, CDAA diet containing 0.05% DL-ethionine or rats fed the CDAAE diet; CSAA, CDAA diet supplemented with choline or rats fed the CSAA diet; HCCs, hepatocellular carcinomas; PCR, polymerase chain reaction; PNNs, preneoplastic nodules; RT, reverse transcriptase; SSCP, single-strand conformation polymorphism

### ACKNOWLEDGEMENTS

We wish to express our thanks to Yonglin Ren for valuable technical assistance and to Madelaine Dusseau for secretarial assistance. This research was supported in part by the National Institute of Health USA grant CA23449, by funds from the Pathology Education and Research Foundation, Pittsburgh, PA, USA, and by a grant for Scientific Research Expenses for Health and Welfare Programs, Japan.

### REFERENCES

Christman JK, Chen ML, Sheikhnejad G, Dizik M, Abileah S and Wainfan E (1993) Methyl deficiency, DNA methylation and cancer: studies on the reversibility of the effects of a lipotrope deficient diet. *J Nutr Biochem* **4**: 672–680

Cox R and Farber E (1972) Ethylation of DNA versus cancer induction with ethionine. *Proc Am Assoc Cancer Res* **13**: 97

Farber E (1963) Ethionine carcinogenesis. *Adv Cancer Res* **7**: 383–474

Farber E (1968) Biochemistry of carcinogenesis. *Cancer Res* **28**: 1859–1869

Ghoshal AK, Sarma SR and Farber E (1986) Ethionine in the analysis of the possible roles of methionine and choline deficiencies in carcinogenesis. In *Essential Nutrients in Carcinogenesis*, Poirier LA, Newberne PM and Pariza MW. (eds), pp. 283–292. Plenum Press: New York

Greenblatt MS, Bennet WP, Hollstein M and Harris CC (1994) Mutations in the p53 tumor suppressor gene: clues to cancer etiology and molecular pathogenesis. *Cancer Res* **54**: 4855–4878

Harvey DM and Levine AJ (1991) p53 alterations is a common event in the spontaneous immortalization of primary BALB/C murine embryo fibroblasts. *Genes Dev* **5**: 2375–2385

Hollstein M, Sidransky D, Vogelstein B and Harris CC (1991) p53 mutations in human cancers. *Science* **253**: 49–53

Kawasaki ES and Wang AM (1989) Detection of gene expression. In *PCR Technology: Principles and Applications*, Erlich HA. (ed.), pp. 89–104. Stockton Press: New York

Leopold WR, Miller JA and Miller EC (1982) Comparison of some carcinogenic, mutagenic, and biochemical properties of S-vinylhomocysteine and ethionine. *Cancer Res* **42**: 4364–4374

Lombardi B and Smith ML (1994) Tumorigenesis, protooncogene activation, and other gene abnormalities in methyl deficiency. *J Nutr Biochem* **5**: 2–9

McArthur CS and Lucas CC (1950) Oral toxicity and lipotropic potency of the triethyl homologue of choline. *Biochem J* **46**: 226–231

McArthur CS, Lucas CC and Best CH (1947) The mode of action of lipotropic agents. Proof of the in vivo incorporation of triethyl-beta-hydroxyethyl-ammonium hydroxide into the phospholipid molecule. *Biochem J* **41**: 612–618

Nakae D, Yoshiji H, Maruyama H, Kinugasa T, Denda A and Konishi Y (1990) Production of both 8-hydroxydeoxyguanosine in liver DNA and γ-glutamyl transferase-positive hepatocellular lesions in rats given a choline-deficient, L-amino acid-defined diet. *Jpn J Cancer Res* **81**: 1081–1084

Nakae D, Yoshiji H, Mizumoto Y, Horiguchi K, Shiraiwa K, Tamura K, Denda A and Konishi Y (1992) High incidence of hepatocellular carcinomas induced by a choline L-amino acid defined diet in rats. *Cancer Res* **52**: 5042–5045

Nakae D, Mizumoto Y, Yoshiji H, Andoh N, Horiguchi K, Shiraiwa K, Kobayashi E, Endoh T, Shimoji N, Tamura K, Tsujiuchi T, Denda A and Konishi Y (1994) Different roles of 8-hydroxyguanine formation and 2-thiobarbituric acid-reacting substance generation in the early phase of liver carcinogenesis induced by a choline-deficient, L-amino acid-defined diet in rats. *Jpn J Cancer Res* **85**: 499–505

Newberne PM (1986) Lipotropic factors and oncogenesis. In *Essential Nutrients in Carcinogenesis*, Poirier LA, Newberne PM and Pariza MW. (eds), pp. 223–251. Plenum Press: New York

Orita M, Suzuki Y, Dekiya T and Hayashi K (1989) Rapid and sensitive detection of point mutations and DNA polymorphisms using the polymerase chain reaction. *Genomics* **5**: 874–879

Poirier LA (1986) The role of methionine in carcinogenesis in vivo. In *Essential Nutrients in Carcinogenesis*, Poirier LA, Newberne PM and Pariza MW. (eds), pp. 269–282. Plenum Press: New York

Polisson C and Morgan RD (1990) AciI, a unique restriction endonuclease from *Arthrobacter citreus* which recognizes 5′ CCGC 3′. *Nucleic Acids Res* **18**: 5911

Rand A, Glenn KS, Alvares CP, White MB, Thibodeau SM and Karnes Jr WE (1996) p53 functional loss in a colon cancer cell line with two missense mutations (218leu and 248trp) on separate alleles. *Cancer Lett* **98**: 183–191

Saito R, Jahnke-Spinnenweber E, Shinozuka H and Lombardi B (1994) On the role of compensatory mitogenesis in the hepatocarcinogenicity of choline and multiple-lipotrope devoid diets. *Carcinogenesis* **15**: 1413–1419

Sambrook J, Fritsch EF and Maniatis T (1989) *Molecular Cloning: A Laboratory Manual*, 2nd edn. Cold Spring Harbor Laboratory Press: Cold Spring Harbor, New York

Shinozuka H, Lombardi B, Sell S and Iammarino RM (1978) Enhancement of ethionine liver carcinogenesis in rats fed a choline-deficient diet. *J Natl Cancer Inst* **61**: 813–817

Shinozuka H, Katyal SL and Perera MIR (1986) Choline deficiency and chemical carcinogenesis. In *Essential Nutrients in Carcinogenesis*, Poirier LA, Newberne PM and Pariza MW. (eds), pp. 253–267. Plenum Press: New York

Smith ML, Yeleswarapu L, Scalamogna P, Locker J and Lombardi B (1993) p53 mutations in hepatocellular carcinomas induced by a choline-devoid diet in male Fischer 344 rats. *Carcinogenesis* **14**: 503–510

Soussi T, Caron DE Fromentel C, Breugnot C and May E (1988) Nucleotide sequence of a cDNA encoding the rat p53 nuclear oncoprotein. *Nucleic Acids Res* **16**: 11384

Swann PF, Pegg AE, Hawks A, Farber E and Magee PN (1971) Evidence for ethylation of rat liver deoxyribonucleic acid after administration of ethionine. *Biochem J* **123**: 175–181

Tsujiuchi T, Kobayashi E, Nakae D, Mizumoto Y, Andoh N, Kitada H, Ohashi K, Fukuda T, Kido A, Tsutsumi M, Denda A and Konishi Y (1995) Prevention by methionine of enhancement of hepatocarcinogenesis by coadministration of a choline-deficient L-amino acid defined diet and ethionine in rats. *Jpn J Cancer Res* **86**: 1136–1142

© Cancer Research Campaign 1997 

Vancutsem PM, Lazarus P and Williams GM (1994) Frequent and specific mutations of the rat p53 gene in hepatocarcinomas induced by tamoxifen. *Cancer Res* **54**: 3864–3867

Vogelstein B and Kinzler W (1992) Carcinogens leave fingerprints. *Nature* **355**: 209–210

Weghorst CM, Buzard GS, Calvert RJ, Hulla JE and Rice JM (1995) Cloning and sequence of a processed p53 pseudogene from rat: a potential source of false 'mutations' in PCR fragments of tumor DNA. *Gene* **166**: 317–322

Ziegler A, Jonason AS, Leffel DJ, Simon JA, Sharma HW, Kimmelman J, Remington L, Jacks T and Brash DE (1994) Sunburn and p53 in the onset of skin cancer. *Nature* **372**: 773–776

*© Cancer Research Campaign 1997*